# Learning pathology using collaborative vs. individual annotation of whole slide images: a mixed methods trial

**DOI:** 10.1186/s12909-016-0831-x

**Published:** 2016-12-12

**Authors:** Michael Sahota, Betty Leung, Stephanie Dowdell, Gary M. Velan

**Affiliations:** Department of Pathology, School of Medical Sciences, Faculty of Medicine, UNSW Australia, Sydney, Australia

**Keywords:** Collaborative learning, Virtual microscopy, Annotation, eLearning

## Abstract

**Background:**

Students in biomedical disciplines require understanding of normal and abnormal microscopic appearances of human tissues (histology and histopathology). For this purpose, practical classes in these disciplines typically use virtual microscopy, viewing digitised whole slide images in web browsers. To enhance engagement, tools have been developed to enable individual or collaborative annotation of whole slide images within web browsers. To date, there have been no studies that have critically compared the impact on learning of individual and collaborative annotations on whole slide images.

**Methods:**

Junior and senior students engaged in Pathology practical classes within Medical Science and Medicine programs participated in cross-over trials of individual and collaborative annotation activities. Students’ understanding of microscopic morphology was compared using timed online quizzes, while students’ perceptions of learning were evaluated using an online questionnaire.

**Results:**

For senior medical students, collaborative annotation of whole slide images was superior for understanding key microscopic features when compared to individual annotation; whilst being at least equivalent to individual annotation for junior medical science students. Across cohorts, students agreed that the annotation activities provided a user-friendly learning environment that met their flexible learning needs, improved efficiency, provided useful feedback, and helped them to set learning priorities. Importantly, these activities were also perceived to enhance motivation and improve understanding.

**Conclusion:**

Collaborative annotation improves understanding of microscopic morphology for students with sufficient background understanding of the discipline. These findings have implications for the deployment of annotation activities in biomedical curricula, and potentially for postgraduate training in Anatomical Pathology.

**Electronic supplementary material:**

The online version of this article (doi:10.1186/s12909-016-0831-x) contains supplementary material, which is available to authorized users.

## Background

Histology and histopathology are the studies of microscopic morphology of normal and abnormal tissues, respectively. Traditionally, the learning and teaching of both histology and histopathology required the use of glass slides and light microscopes. Students would examine slides and attempt to compare what they saw down their microscope to the examples provided by their instructor. In the context of increasing class sizes in medical schools, the traditional model of learning histology and histopathology using light microscopy (LM) has become impractical.

Virtual microscopy (VM) is the use of computer technology to view digitised versions of glass slides as whole slide images (WSIs) [1]. To enable this approach, WSIs are typically served via the Internet and viewed with a web browser. An important advantage of VM is that it allows access to WSIs outside of scheduled class times [1–5]. It has been demonstrated that students access VM materials throughout the day [6]. Additionally, VM may also enhance students’ learning [7, 8]. However, it is worth noting that VM can be expensive to set up and is prone to technical difficulties [1–4, 8–10].

As students view identical tissue sections using VM, the risk of variability in learning materials is eliminated [1, 2, 4, 8, 11–13]. However, this may promote the tendency for students to undervalue the importance/existence of variation in tissue sections [14]. It has been suggested by Helle and colleagues [15] that learning using virtual microscopy results in greater learning gains for high-performing students, compared with lower-performing students. However, aggregated analysis of all participants in that study showed no significant difference between students learning with VM and LM.

In contrast to the studies promoting the benefits of VM for learning, several trials comparing VM with traditional methods of learning histology and histopathology found no net improvement in student performance [1, 6, 10, 16–19]. These findings are unsurprising, because activities that promote interaction and engagement with microscopic morphology are needed to improve learning outcomes, irrespective of the medium employed. Nevertheless, a meta-analysis of virtual microscopy using multiple standardised comparative studies concluded that, on balance, VM is superior to LM for learning [14].

From 2002, UNSW Australia (UNSW) transitioned from using LM to VM in histology and pathology classes. This innovation significantly enhanced students’ learning experiences [18]. Each year over 3000 biomedical students in UNSW undertake 100,000 student hours of practical work in subjects that require an understanding of microscopic appearances of human tissue. As a member of the Biomedical Education Skills and Training (BEST) Network established in 2013, UNSW adopted the use of Slice™ [20], a biomedical image repository and image viewer, to enhance the efficiency and availability of viewing WSIs.

VM has enabled the establishment of ‘digital laboratories’, which allow students and teachers to build unique and personalised learning materials, i.e. annotations [3–6, 8, 12, 21]. Such annotations may enhance learning by promoting longer and more meaningful interactions between students and learning resources [21].

**Fig. 1:**
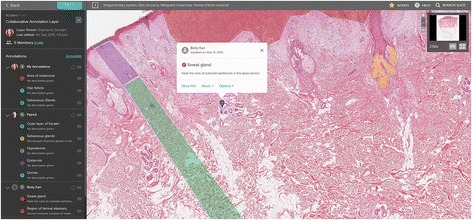
Screenshot showing the interface and capabilities of Slice for annotation of WSIs. The ability to annotate and invite others to the annotation layer is shown via the blue links on the *top left* of the figure. Users of the collaborative layer and their respective annotations are shown in a list to the left of the figure. All annotations in the current viewing area (indicated by the navigation box at the *top right*) are represented simultaneously on the screen (*centre*)

Recently, the functionality of Slice was enhanced to enable users to collaboratively annotate WSIs in real-time (Fig. 1). This facilitates the implementation of collaborative learning activities involving both students and teachers. Using Slice, learners can collaborate by sharing annotations on a common image layer using any device, from any location.

Collaborative learning is an educational methodology that focuses upon the interactions between participants in the learning process, creating a sense of community. Specifically, a sense of community promotes opportunities for active engagement and interaction, which in turn result in improved levels of self-perceived learning, skill enhancement, enjoyment, engagement and other learning outcomes for individuals [22–26]. Furthermore, factors such as group dynamics and student perceptions of learning can potentiate the learning outcomes of collaboration [27, 28].

It is therefore unsurprising that collaborative learning modalities have been globally employed across many different fields. Collaborative approaches have been progressively incorporated into virtual environments that have been shown to further improve students’ engagement and learning outcomes [29–34]. Such forms of learning are categorised as computer-supported collaborative learning (CSCL) environments. By utilising computers, more avenues of achieving and displaying collaborative interaction are available for exploration [35].

Additionally, CSCL environments can aid in the navigation of complex tasks by reducing cognitive load. This is achieved by enabling students to focus on sub-elements to address a bigger and more complex problem [36]. In such socio-constructivist environments, the role of the teacher changes from a dispenser of knowledge to a facilitator of knowledge exchange and co-creation.

Thus, students readily accept both VM and collaborative learning approaches. The existing literature indicates that both approaches independently benefit student learning. However, very few studies evaluate the qualitative and quantitative impact on student learning of collaborative learning using VM [14].

Implementing a virtual collaborative learning environment has been shown to result in improved learning efficiency for students with no degradation in summative examination performance. Students also provided positive feedback regarding the ‘opportunity to collaborate’ when utilising VM [5, 6, 16, 19, 37–40]. However, one of the rare studies that described collaborative annotations on WSI [6], while demonstrating enhanced engagement by students, did not show any improvement in learning outcomes.

Thus, it remains to be determined whether collaborative learning using VM is significantly better for knowledge acquisition than individual annotation. The present study aimed to evaluate the quantitative and qualitative differences in learning with individual and collaborative annotation of WSIs for cohorts of junior and senior students.

## Methods

### Participants

Students enrolled in Medicine and Medical Science programs at UNSW were recruited to participate in this study during their Pathology practical classes. The student cohorts were:

1. Junior students in Year 2 of a Bachelor of Medical Science program, who were enrolled in an introductory Pathology course (*n* = 119);
2. Senior students in the enrolled in a selective course known as Rational Use of Investigations in the final year (Year 6) of the Medicine program (*n* = 12).

These students were chosen for this study because they represent novices (Year 2 Medical Science) and experienced users of virtual microscopy in Pathology respectively.

Students were advised that they could opt-out of study participation at any time. To encourage participation, the annotation activities were integrated into existing class structures.

### Trial design

Formal instruction and regular classwork occurred within the first half of each 2-h Pathology practical classes. For the remainder of each class, the sequential assessment of individual and collaborative annotation activities took place. All students in each class annotated a set of WSIs under the same initial (either collaborative or individual) conditions. The entire class then crossed over to the alternate condition (either individual or collaborative) to annotate a second set of WSI. The initial condition alternated between consecutive iterations of the same class, while identical sets of WSIs were used (Table 1). This protocol aimed to control for potential carry-over effects, whereby the order of individual and collaborative annotation activities might have affected quiz performance. Annotation activities were 30 min in duration. No formal feedback was provided to students during the intervention. However, following the intervention, all students received automated feedback in their performance in timed online quizzes.

**Table 1:** Sequential Cross-Over Trial Structure

	Class 1	Class 2
WSI 1 (Slides A & B)	Collaborative	Individual
30 min of normal classwork		
WSI 2 (Slides C & D)	Individual	Collaborative
5-min questionnaire		
10-min online quiz		

The trials for junior medical science students enrolled in an introductory course in Pathology were conducted with four WSIs. The first class (Class 1, *n* = 63) completed individual annotation activities on WSIs showing a tubular adenoma of the colon and a colorectal carcinoma. After a brief 30-min break from intervention conditions (washout period) consisting of traditional classwork, the class then completed collaborative annotation activities on WSIs showing squamous cell carcinoma of the tongue and invasive ductal carcinoma of the breast. All students then individually completed the questionnaire and a timed quiz online.

The second junior class (Class 2, *n* = 56) replicated the same order of activities as Class 1, but annotated the WSIs showing tubular adenoma and the colorectal adenocarcinoma collaboratively, then proceeded to individually annotate the WSIs showing squamous cell carcinoma of the tongue and invasive ductal carcinoma of the breast. These students then completed the same questionnaire and timed quiz as students in Class 1.

The trial for senior medical students enrolled in a Pathology selective course also utilised four WSIs. The class first annotated a WSI showing diffuse alveolar damage individually. After a brief washout period of traditional classwork, students then collaboratively annotated a WSI showing a pulmonary carcinoid tumour. All students then completed the online questionnaire and a timed quiz on the features of relevant WSI.

The same sequential cross-over protocol was replicated with the same group of students 1 week later, this time employing WSIs showing endometrial adenocarcinoma and herpes oesophagitis, followed by a timed quiz assessing those topics.

These tasks were supported by written and spoken task descriptions, a tailored digital learning environment in which to perform the annotation tasks and instructor feedback on student performance at the end of the task. Task control factors refer to the extent to which learners can control the task - specifically, the path, pace, content and instruction. Student task control in this study was limited due to time and classroom constraint considerations.

### Annotation activities

Once allocated to either individual or collaborative annotation conditions, students were asked to annotate WSIs, focusing on a small number of selected features. These activities were designed to provide visual cues to assist students’ understanding of the microscopic features of disease processes. Such visual cues trigger the interpretive process, resulting in improved pattern recognition, student performance, productivity and efficiency on diagnostic tasks [5, 38, 41].

#### Collaborative annotation

Students allocated to the collaborative annotation condition were asked to identify microscopic features by annotating WSIs in randomly self-allocated groups of 3–5 students. This number of students working collaboratively was both logistically suitable and in accordance with existing literature [16]. Students engaged in collaborative annotation activities could view the annotations of their peers, thus enabling them to review and update their own annotations based upon peer feedback.

A CSCL environment was employed to facilitate conditions under which students would be more likely engage in collaboration with peers to improve learning outcomes, i.e. engage in discussion, negotiation & self-regulation. This was achieved by each students creating digital artefacts (annotations) from their own computer, then comparing their annotations in real-time to those created by their peers. The primary collaborative element of the intervention is the ability for students to edit their own annotations based upon the seeing and discussing the annotations made by their peers. By influencing and learning from one another’s annotations, students were then able to produce a final product or ‘consensus annotation’ based on the shared understanding of the group.

#### Individual annotation

When allocated to the individual annotation condition, students were asked to work alone to identify and annotate pre-selected histological features of a set of WSIs. During this period, students could not view the annotations that their peers created and except for technical assistance, instructor feedback was minimised.

Students who were working individually did not have access to the annotations created by their peers, nor did they have access to peer-based discussion outside of the information provided to both groups by the instructor.

#### Whole slide image selection

The WSIs chosen for the study were derived from the curriculum pertaining to each student cohort and were accessed via the Slice™ image database [20]). All WSIs selected for this study had not been previously examined by the students, and thus contained novel material. WSIs and associated annotation activities were reviewed by subject matter experts to ensure that the level of difficulty was suitable for each cohort.

### Evaluation of knowledge and perceptions of learning

#### Design of online quizzes

**Fig. 2:**
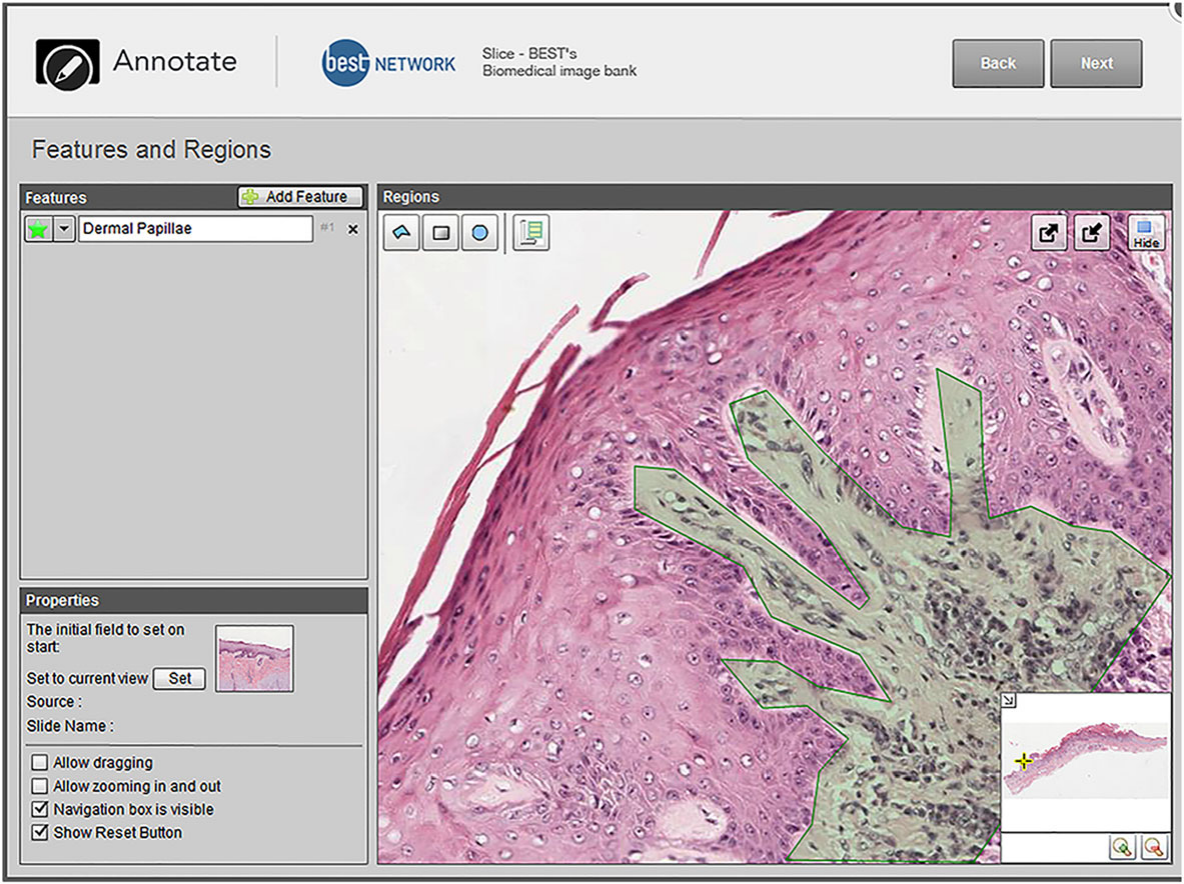
Example of the authoring environment for creation of a drag and drop question using the Adaptive eLearning Platform

After completing both individual and collaborative annotation activities, participants in each cohort attempted tailored 10-min time-limited online quizzes, created using the Adaptive eLearning Platform (AeLP) developed by Smart Sparrow Ltd (Sydney, Australia) [42]. Quizzes were linked securely from the university’s Learning Management System. The quizzes for each cohort related to the WSIs explored during the preceding annotation activities. There were a total of nine items in the junior quiz, and 13 items in the quizzes for senior students. Items included feature identification using drag and drop (Fig. 2) and drop-down lists, as well as image-based multiple choice items. These questions primarily focused on the correct identification of histopathological features of specific disease entities by utilising previously unseen WSI showing the same pathological processes explored in class.

To minimise bias in favour of either the individual or collaborative annotation conditions, subject matter experts designed and reviewed all quiz items to ensure that they were of comparable difficulty, and were appropriate for the level of the students [43]. To facilitate comparisons of performance between conditions, care was taken to ensure that all quizzes contained an equal number of items related to each WSI, and that all items were of similar difficulty. Thus, students in each cohort were given equal opportunity to display their understanding of microscopic features studied either collaboratively or individually.

#### User experience questionnaire

To evaluate students’ perceptions of their learning experience using WSI annotation, an online questionnaire was developed. The questionnaire items had previously been validated as part of the Perceived Utility of Learning Technologies Scale (G. Velan, personal communication) and were employed to gather student feedback regarding the Slice platform, individual and collaborative annotation activities, as well as student perceptions of understanding the microscopic features of diseases, before and after annotation. The questionnaires were presented to participants online, immediately preceding the timed quiz. This order was employed to maximise the likelihood of participants completing the questionnaire.

### Data analysis

Quiz and questionnaire data were extracted from the AeLP as comma delimited text files, which were opened with Microsoft Excel® (Microsoft Software Inc., Redmond, Washington). The data was de-identified and sorted before being imported into GraphPad Prism 6 v6.04 (GraphPad Software Inc., San Diego, California) for statistical analysis.

#### Quantitative analysis

##### Quiz data

Comparisons between groups of students within the same cohort were performed using unpaired t-tests.

Comparisons of individual students’ performance in quiz questions related to the collaborative and individual annotation conditions were performed using paired t-tests.

All quiz data are expressed as mean percentage scores ± 95% confidence interval (CI). Differences between groups or conditions were considered statistically significant when the *p*-value was observed to be less than 0.05. When significant differences were detected, effect sizes were calculated using Cohen’s d.

##### Questionnaire data

Comparisons of students’ perceived understanding before and after collaborative annotation activities were performed using a Mann-Whitney U test.

Data derived from Likert scale items and the ranking item in the questionnaire are expressed as median ± interquartile range (IQR).

#### Qualitative analysis

Responses to open-ended questionnaire questions were analysed using a grounded theory approach [44, 45]. Briefly, this involved the collection and review of qualitative data such that repeated ideas, concepts and themes were codified and then grouped into overarching themes that were then characterised as either positive or negative. Saturation was deemed to have been achieved when no new codes were detected. The analysis was performed independently by two investigators, and any differences were resolved by consensus.

## Results

### Quantitative results

#### Class equivalency

In order to determine the equivalence of two classes of second-year medical science students, overall quiz results were compared. There was no statistically significant difference in mean quiz performance between classes (Class 1 *n* = 63, mean = 57.53%, 95% CI = 54.00–61.05%; Class 2 *n* = 56, mean = 60.74%, 95% CI = 57.35–64.14%; *t* = 1.295, df = 335; *P* = 0.1961). Quiz results for all students in that cohort were therefore pooled for further analyses.

#### Quiz scores: individual vs collaborative

Junior students’ mean quiz scores following individual annotation did not differ significantly from mean quiz scores following collaborative annotation—(individual mean = 61.51%, 95% CI = 57.54–65.49%; collaborative mean = 57.98%, 95% CI = 53.62–62.34%; *t* = 1.549, df = 118; *P* = 0.1241; *n* = 119).

Amongst the senior (Phase 3 Medicine) cohort, there was a statistically significant difference in favour of collaborative annotation over individual annotation, with a large effect size (Cohen’s d = 1.37) (individual mean = 57.58%, 95% CI = 47.32–67.83%; collaborative mean = 77.78%, 95% CI = 69.53–86.02%; *t* = 3.416, df = 11; *P* = 0.006; *n* = 12).

#### Students’ perceptions of understanding

There was a statistically significant improvement between students’ median perceptions of understanding on a scale from 1 to 10 before and after annotation for junior (median before annotation = 4 out of 10, interquartile range 2–5; median after annotation = 6 out of 10, interquartile range 3–7; *P* < 0.0001; *n* = 119) and senior students (median before annotation = 3.5 out of 10, interquartile range 2.25–5; median after annotation = 7 out of 10, interquartile range 5.25–8; *P* = 0.005; *n* = 12).

From the questionnaire data, median responses to the “Learning Effectiveness through Collaboration” item were significantly higher for the senior cohort (Junior median 4, interquartile range 3–5; Senior median 5, interquartile range 4–6; *P* = 0.04).

### Qualitative results

#### Positive feedback

Junior students’ perceptions of learning using the annotation activities were obtained from their open-ended questionnaire responses. Positive feedback comments (*n* = 73) from these students indicated that the ability to annotate (*n* = 36), the user-friendly Slice™ interface (*n* = 22), improved understanding (*n* = 19), collaboration with peers (*n* = 17) and the engagement/interactivity provided by the activities (*n* = 15) were the most enjoyable aspects of the intervention.

For senior students, positive comments (*n* = 9) focused on the ability to annotate (*n* = 7), opportunity to collaborate (*n* = 1) and level of engagement/interaction (*n* = 1).

Representative positive feedback comments from each cohort are shown in Table 2 (for full feedback see Additional files 1 and 2).

**Table 2:** Representative positive feedback comments from each cohort

Representative Positive Feedback		Theme Identified
Junior	*Being able to annotate on the slide itself, as well as being able to share slides with colleagues. High quality virtual slides are also better than textbook or lecture slides.*	Annotation; Collaboration; Interface
	*I enjoyed the group component of it. The discussion helped facilitate my understanding of the topic.*	Collaboration; Improved Understanding;
	*Collaboration allows me to pull on resources that I do not normally have in order to gain a better understanding of the topic.*	Collaboration; Improved Understanding;
Senior	*Annotation and the ability to collaborate*	Annotation; Collaboration;
	*Ease of use. Engaging way of arranging learning activities*	Interface Engaging;

#### Negative feedback

Negative feedback on the annotation activities was also gathered from open-ended questionnaire responses. Junior students’ negative comments (*n* = 82) indicated concerns with insufficient guidance/feedback (*n* = 30), insufficient integration of the intervention into the class (*n* = 20), lack of prior knowledge to make best use of the collaborative activities (*n* = 6) and issues with the technology (*n* = 6).

Senior students’ negative feedback (*n* = 7) focused on a need for more guidance/feedback (*n* = 4) as well as the technological limitations of the software (*n* = 3).

Representative negative feedback by cohort is shown in the Table 3 (for full feedback see Additional files 1 and 2).

**Table 3:** Representative negative feedback comments from each cohort

Representative Negative Feedback from Junior Students		Theme Identified
Junior	*I would like if answers could be provided for the activity after we have conducted our own team or individual attempt, that way we can see whether our understanding is correct.*	Lack of feedback
	*More background information e.g. an image of a normal structure and orientation on things that we should be looking out for. Some annotation activities are particularly difficult without this, e.g. obscure tissue like breasts that we don’t often look at the histology of.*	Not enough background knowledge
	*It would be nice if there was a way for everyone in a group to be able to edit annotations/layers simultaneously because that would make it a lot easier to contribute information and receive other answers.*	Interface
Senior	*Once we start annotating the instructions are no longer available.*	Interface
	*To enhance self-study, perhaps some pre-annotated slices with features marked and explained.*	Lack of feedback

## Discussion

For senior students in this study, collaborative WSI annotation resulted in significantly improved quiz scores when compared with individual WSI annotation. In contrast, there were no significant differences in quiz scores between collaborative and individual annotation conditions for junior students.

The above findings align with previous studies, which demonstrated that students with greater amounts of knowledge and experience are more likely to benefit from the collaborative learning process [26, 27, 46–48]. While this phenomenon is likely to be primarily related to the extent of students’ background knowledge of the discipline area, there may be other contributory factors. Specifically, as opposed to junior students, senior students’ extensive history of socialising and collaborating is likely to have positively influenced their interactions with one another, potentiating their collaborative learning outcomes [28]. Furthermore, in comparison to many junior Medical Science students, these senior medical students were high-achieving and highly motivated– such learners have been shown to benefit more from VM-based collaboration compared to their lesser-achieving counterparts [15, 27].

Both cohorts of students perceived significant improvements in their understanding following participation in the annotation activities. This finding was expected as collaborative WSI annotation was designed to increase student engagement, which is positively correlated with perceptions of improved understanding [3–5, 7, 8, 12, 38, 41, 47, 48].

It is noteworthy that senior students perceived collaborative annotation to be significantly more effective for learning than the junior cohort. Nevertheless, both cohorts perceived significant improvements in their understanding following annotation activities. The discrepancy between self-assessment of understanding and quiz performance by the junior cohort in this study might be related to a lack of regular self-assessment by students [49, 50]. This, together with deliberate withholding of teacher feedback prior to the quiz, could have affected the accuracy of junior students’ self-assessment [51].

Themes that recurred across each cohort regarding students’ positive perceptions of annotation activities on WSIs included collaborating with peers, as well as increased interactivity and engagement. These aspects are consistent with previous studies of students’ learning preferences, that is by interacting and engaging with learning materials in a social environment [5, 6, 16, 19, 37–40, 52].

The negative feedback responses gathered from the open-ended questionnaire focussed primarily on insufficient feedback and technical issues. The reported lack of feedback is understandable, because all formal feedback was withheld from students prior to the online quiz to avoid biasing quiz outcomes. Criticisms of the Slice™ platform’s functionality have been utilised to inform further developments.

### Limitations

For senior students (*n* = 12), this study was underpowered. Nevertheless, this study demonstrated a statistically significant difference in quiz scores in favour of collaborative annotation for the senior cohort, with a large effect size. This is indicative of an important real-world difference in learning between individual and collaborative conditions for senior students.

It might have been helpful to employ a pre-annotation test of understanding of microscopic Pathology for each cohort, in order to better quantify the improvement procured from the annotation exercises. However, such pre-tests were not logistically possible, and may even have biased the results of the study, via test-enhanced learning [53].

Furthermore, the intervention was limited by the number and scope of WSI that were used for annotation and assessment purposes, i.e. two WSI per condition, four per cohort. While it might have been helpful to create more data points, we were constrained by the time available in each class.

The extent to which students could control their learning was limited by instructors, i.e. the path, overall pace, content and instruction was predetermined by instructors and was subject to classroom and time constraints [54]. In this sense, the learning environment was scaffolded and was not a truly free CSCL environment.

Participants were informed that no course credit would be awarded for their performance in the knowledge quizzes. Therefore, lack of motivation to succeed might have affected students’ performance in the quizzes. However, these factors are unlikely to have biased results in favour of either individual or collaborative annotation conditions.

A risk inherent in cross-over studies is the potential for carryover effects following the cross over, which can reduce observed differences between groups. In particular, it is possible that those students who commenced with collaborative annotation might have been advantaged in subsequent individual annotation activities. In our sequential cross-over design, carryover effects were controlled for by alternating the order of individual and collaborative annotation activities between classes. Within each cohort, analyses of quiz performance showed no significant difference between classes, thereby providing reassurance that carryover effects did not bias the outcomes of this study. A washout period, such as that provided in this study, might also have helped to reduce such carryover effects.

## Conclusions

This is the first reported study that has critically evaluated student knowledge acquisition using collaborative annotation of WSI. The mix of quantitative and qualitative methods utilised in this study provided a realistic overall picture of student learning in that context. For senior medical students in this study, collaborative WSI annotation was superior for understanding key microscopic features when compared to individual WSI annotation. Collaborative annotation was equivalent to individual annotation for junior medical science students. These findings have implications for the deployment of annotation activities in Medicine and Medical Science, as well as a variety of other disciplines that utilise images to facilitate the learning of morphology.

This investigation showed that students positively perceive collaborative learning, regardless of experience level. However, in the discipline of histopathology, collaborative annotation of WSIs was shown to be objectively beneficial only for senior students with sufficient background knowledge and experience.

### Future research

It might be beneficial to replicate this study, while correlating students’ prior academic performance with their quiz scores. This would enable exploration of whether high-performing students benefit more or less from a collaborative approach than low-performing students, as has been suggested previously [15]. The administration of a pre-intervention test would also provide a baseline level for comparison with the post-intervention results [55].

Studies to evaluate knowledge retention rates over time may be valuable in providing a longitudinal view of student learning. Such studies might provide crucial evidence of the long-term benefit of collaborative annotation of WSIs.

Finally, collaborative annotation on WSIs might have potential to optimise learning for Anatomical Pathology trainees. If further studies in such settings validate the positive impact of collaborative annotation, this could have implications for specialist training in Anatomical Pathology.

## Additional files

Additional file 1: Junior student raw data. This spreadsheet contains all of the raw quiz score and qualitative data obtained from junior medical science students. This data underpins the findings in this report. (XLS 395 kb)
Additional file 2: Senior student raw data. This spreadsheet contains all of the raw quiz score and qualitative data obtained from senior medicine students. This data underpins the findings in this report. (XLS 52 kb)

## Abbreviations

AeLP: Adaptive eLearning Platform
BEST: Biomedical Education Skills & Training
CI: Confidence interval
CSCL: Computer-supported collaborative learning
IQR: Interquartile range
LM: Light microscopy
UNSW: University of New South Wales
VM: Virtual microscopy
WSI: Whole slide image
